# Dietary Flavonoids and the Risk of Colorectal Cancer: An Updated Meta-Analysis of Epidemiological Studies

**DOI:** 10.3390/nu10070950

**Published:** 2018-07-23

**Authors:** Hui Chang, Lin Lei, Yun Zhou, Fayin Ye, Guohua Zhao

**Affiliations:** College of Food Science, Southwest University, Chongqing 400715, China; changhui2017@swu.edu.cn (H.C.); leilinsky@hotmail.com (L.L.); zhouy2017@swu.edu.cn (Y.Z.); fye@swu.edu.cn (F.Y.)

**Keywords:** dietary flavonoids, flavonoid subclass, flavonols, colorectal cancer, meta-analysis

## Abstract

**Aim**: The aim of this study was to perform an up-to-date meta-analysis of the association between the intake of dietary flavonoids and the risk of colorectal cancer. **Methods**: The PubMed and EMBASE databases were searched to identify eligible studies. The risk of colorectal cancer for the highest versus the lowest categories of flavonoids intake were assessed. **Results**: A total of 12 studies (5 cohort and 7 case-control studies) involving 17,481 cases and 740,859 controls were eligible for meta-analysis. High intake of dietary flavonols, flavones and anthocyanidins may decrease the risk of colorectal cancer; the pooled odds ratio (OR) for the highest intake compared with the lowest was 0.70 (0.54–0.90), 0.79 (0.83–0.99) and 0.78 (0.64–0.95), respectively. No association between the intake of total flavonoids, flavanones or flavan-3-ols and the risk of colorectal cancer was observed. Furthermore, the data showed that high intake of flavonols may decrease the risk of colon cancer [0.80 (0.68–0.94)] but not rectal cancer [0.93 (0.74–1.18)], while on the contrary, the intake of flavones may decrease rectal cancer risk [0.82 (0.70–0.97)] but not colon cancer risk [0.88 (0.69–1.13)]. **Conclusions**: The present study suggested that high intake of flavonols (such as quercetin) may reduce the risk of colon cancer, and high intake of flavones (such as apigenin) may reduce the risk of rectal cancer.

## 1. Introduction

Flavonoids are a major class of dietary polyphenols naturally occurring in plant-based foods and beverages, such as fruits, vegetables, tea, wine and juices. It has been well established that diets rich in plant-based foods can reduce the risk of many kinds of cancer, especially gastrointestinal cancers [1,2,3]. The mechanism by which these foods exert a protective effect against tumourigenesis and the development of cancer is unclear, but one hypothesis is the presence of potentially anticancer phytochemicals [4,5]. In the last two decades, much attention has been given to dietary flavonoids in our food and their proposed chemopreventive bioactivities, especially their anti-carcinogenetic properties with regard to gastrointestinal cancers [6,7,8,9].

More than 5000 plant flavonoids have been identified [10]. Based on their chemical structure, flavonoids can be subclassified into six principal subclasses: flavonols (mainly including quercetin, kaempferol, myricetin and isorhamnetin), flavones (apigenin and luteolin), flavanones (hesperetin and naringenin), flavan-3-ols (catechin, epicatechin, epigallocatechin, epicatechin-3-gallate, epigallocatechin-3-gallate), anthocyanins (cyanidin, delphinidin, malvidin, pelargonidin, petunidin, peonidin), and isoflavones (genistein and daidzein). Dietary flavonols mainly exist in tea, onions, broccoli, and various common fruits. Flavanones and flavones are in oranges and other citrus fruits or citrus juice. Flavones are also abundant in vegetables, such as celery, peppers and lettuce. Flavan-3-ols are in green tea, apples, cocoa, red wine, grapes, and other fruits. Anthocyanidines are in coloured berries, black currants, grapes and some vegetables, such as eggplant and radishes. Unlike other flavonoids, isoflavones are mostly contained in soy products instead of fruits, vegetables and tea.

Epidemiological studies and systematic analyses have showed the intimate association between dietary factors and cancer risk. It is interesting and important to elucidate the association between flavonoid intake and the risk of cancers [11,12,13]. In fact, many studies have been performed to estimate the relationship between dietary flavonoid intake and various types of cancer, including colorectal cancer. However, studies have reported inconsistent findings for flavonoid intake and colorectal cancer risk. In the past two years, several population-based studies focused on the association between dietary flavonoid intake and colorectal cancer risk have been published [14,15,16,17]. In particular, a large-scale prospective cohort study, the European Prospective Investigation into Cancer and Nutrition (EPIC) study, with a cohort of 477,312 participants in 10 European countries provided important new data on this topic [17]. To derive a more precise estimation of the relationship between the intake of total flavonoids and flavonoid subclasses and the risk of colorectal cancer, we performed an up-to-date meta-analysis to summarize the available evidence from prospective and case-control studies. Given that isoflavones have a special structure and come from specific food sources, and because many reviews have been conducted to assess the association between isoflavone intake and cancer risk, we excluded this topic in the present study.

## 2. Methods

### 2.1. Search Strategy

We conducted a systematic search of the literature published to 15 May 2018 using the Cochrane, PubMed and EMBASE databases. The following search terms were used: “flavonoid”, “flavonol”, “flavone”, “flavanone”, “flavan-3-ols”, “flavanol”, “anthocyanidin”, “polyphenolic compound” and “colorectal cancer”, “colon cancer”, “rectal cancer”. We also performed a manual search using reference lists of original articles and relevant reviews. Only full-length original journal articles were considered and no attempt was made to include abstracts or unpublished studies.

### 2.2. Study Selection

Articles were eligible for the present meta-analysis if they conformed to the following criteria: (i) the study design was a population-based study, including cohort or case-control study; (ii) a relatively complete assessment of flavonoids intake was performed; (iii) the association of flavonoids intake with colorectal cancer risk was specifically evaluated; and (iv) the relative risk (RR), hazard ratio (HR), or odds ratio (OR) and the corresponding 95% confidence interval (95% CI) values were available. In cases in which duplicate reports from the same study were identified, we chose the most recent one. 

### 2.3. Data Extraction

The data from each paper fulfilling the inclusion criteria were extracted carefully by two independent reviewers. The following information from each study was recorded: (i) the first author’s name, publication year and country or city of origin; (ii) the study design (prospective cohort study, case-control study); (iii) the population (numbers of cases and controls); (iv) flavonoids exposure; (v) the RR or values from the most fully adjusted model and their 95% CI values; and (vi) the listed confounders adjusted for in the multivariate analysis. In addition, due to the low incidence of colorectal cancer, the OR was assumed to be the same as the RR, and the summary results were reported as OR for simplicity. 

### 2.4. Statistical Analysis

The summary ORs and corresponding 95% CIs of the included studies were used as a measure to assess the association of flavonoids intake with colorectal cancer risk. The statistical heterogeneity among studies was assessed using the Q test and *I*^2^ statistics. If a statistical difference in heterogeneity existed (*p* < 0.10 or *I*^2^ > 50%), a random-effects model was selected to pool the data; otherwise, a fixed-effects model was used. When statistical heterogeneity was detected, a sensitivity analysis was performed to explore potential sources of heterogeneity, both in the overall pooled estimate and within the subgroups. The potential publication bias was examined by the funnel plot and Egger’s test (*p* < 0.10). All of the analyses were performed using STATA version 14.0 (Stata Corp., College Station, TX, USA). A *p* value < 0.05 was considered to be statistically significant unless otherwise specified. 

## 3. Results

### 3.1. Characteristics of the Included Studies 

**Figure 1:**
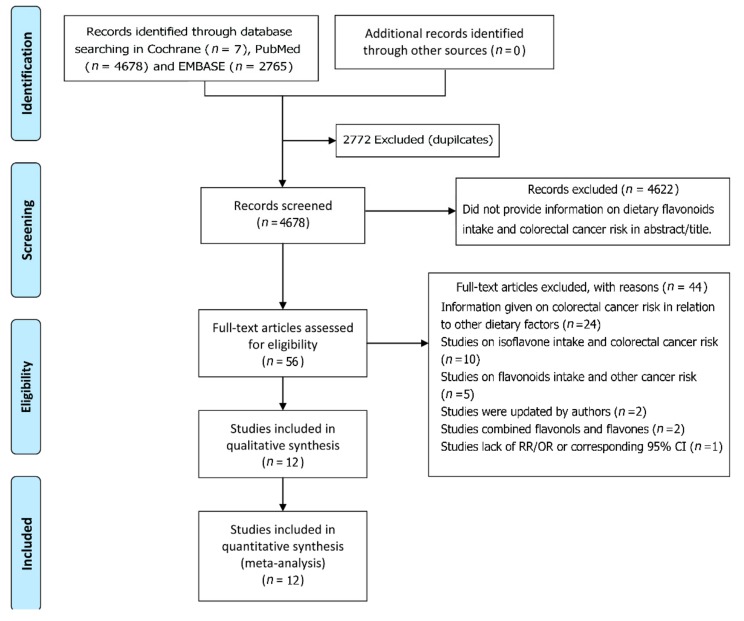
Flow chart showing study selection procedure.

Our systematic search of the literature identified a total of 4678 relevant scientific studies. Among these studies, 4622 irrelevant titles and/or abstracts were excluded, and 56 full-text articles were left for further review (Figure 1). After a more detailed evaluation, 44 studies were excluded as irrelevant or because they did not meet the inclusion criteria. In the end, 12 studies relevant to the association of dietary flavonoids intake with the risk of colorectal cancer were included in the present meta-analysis [14,15,16,17,18,19,20,21,22,23,24,25]. 

The characteristics of the included studies are presented in Table 1. These studies had 5 prospective cohort studies and 7 case-control studies, including 17,481 cases and 740,859 controls. All studies were published in English. Among these 12 studies, 8 were conducted in Europe (2 in UK, 2 in Finland, 1 in Netherland, 1 in Spain, 1 in Italy, 1 in 10 European countries), 2 were conducted in Asia (1 in China and 1 in Korea), and the other 2 studies were in the USA. All studies were adjusted for a wide range of potential confounders of colorectal cancer, such as age, gender, BMI, physical activity, family history of colorectal cancer, education, energy intake, alcohol, fiber intake, red and processed meat intake, tobacco, aspirin, non-steroidal anti-inflammatory drug.

### 3.2. Dietary Flavonoid Intake and the Risk of Colorectal Cancer

**Figure 2:**
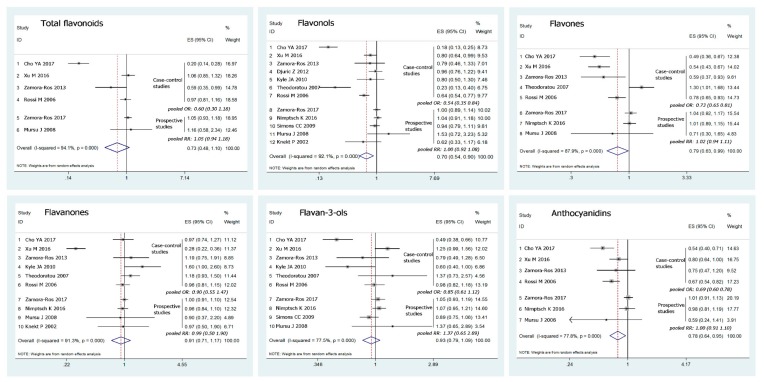
Forest plots of investigating association for flavonoids intake with colorectal cancer risk (highest vs. lowest). Squares indicate study-specific risk estimates (size of the square reflects the study-specific statistical weight, i.e., the inverse of the variance); horizontal lines indicate 95% confidence intervals (CIs); a diamond indicates summary estimate with its corresponding 95% CI.

We calculated the summary OR values of colorectal cancer included in the studies using fixed- or random-effects models, depending on the heterogeneities. As shown in Figure 2, the results indicated that there is no significant association between colorectal cancer risk and total flavonoid intake, with a pooled OR from the combination of the included studies of 0.73 (95% CI: 0.48–1.10) for the highest category of intake vs. the lowest category. Similarly, no association between the intake of flavanones or flavan-3-ols and the risk of colorectal cancer was observed. Meanwhile, our data showed that high intake of dietary flavonols, flavones and anthocyanidins may decrease the risk of colorectal cancer; the pooled ORs for the highest intake compared with the lowest were 0.70 (0.54–0.90), 0.79 (0.83–0.99) and 0.78 (0.64–0.95), respectively. Nevertheless, substantial heterogeneities existed across the studies. 

We estimated ORs of colorectal cancer for different levels of flavonol, flavone and anthocyanidin intake. There was a large variation among studies in the difference in flavonoids intake between the highest and lowest exposure categories (Table 1). To normalize this variability, for each study, we calculated a risk estimate for an increment of flavonols intake of 10 mg per day, flavones intake of 1 mg per day and anthocyanidins intake of 10 mg per day. Dose-response meta-analysis indicated that an increment of dietary flavonols intake of 10 mg per day (pooled OR = 0.86, 95% CI: 0.76–0.97) or flavones intake of 1 mg per day (pooled OR = 0.91, 95% CI: 0.84–0.99) was significantly associated with a deceased risk of colorectal cancer, but anthocyanidins intake of 10 mg per day (pooled OR = 0.93, 95% CI: 0.79–1.07) was not. 

We stratified the included studies by design, sex and population. As shown in Table 2, the pooled RRs for the intake of flavonol, flavone and anthocyanidin for prospective cohort studies were 1.00 (0.92–1.08), 1.02 (0.94–1.11) and 1.00 (0.91–1.10), respectively. Inconsistent with the findings of case-control studies, the prospective cohort studies revealed no significant association between colorectal cancer risk and this intake of flavonoids, and no substantial heterogeneity existed across these studies. Also, the data showed that high intake of flavonols may decrease the risk of colon cancer but not rectal cancer, while the intake of flavones, on the contrary, may decrease rectal cancer risk but not colon cancer risk. Furthermore, statistically significant associations between high intake of these dietary flavonoids and colorectal cancer risk were observed among studies conducted in Asia but not in the USA. In the European population, the intake of flavonols and anthocyanidins, but not flavones, showed a significant association with a decreased risk of colorectal cancer. 

### 3.3. Sensitivity Analysis

Statistical heterogeneities existed across studies in the overall pooled estimate. We performed sensitivity analysis to evaluate the stability of the results, in which individual studies were sequentially dropped. The analysis excluded any single study in turn and pooled the OR of the remaining included studies. The summary OR for total flavonoids and each flavonoid subclass except flavones did not substantially change after excluding any single study. Yet, when we excluded the study in Korean [14], the pooled OR for flavones changed from 0.79 (0.63–0.99) to 0.85 (0.69–1.05). 

### 3.4. Publication Bias

**Figure 3:**
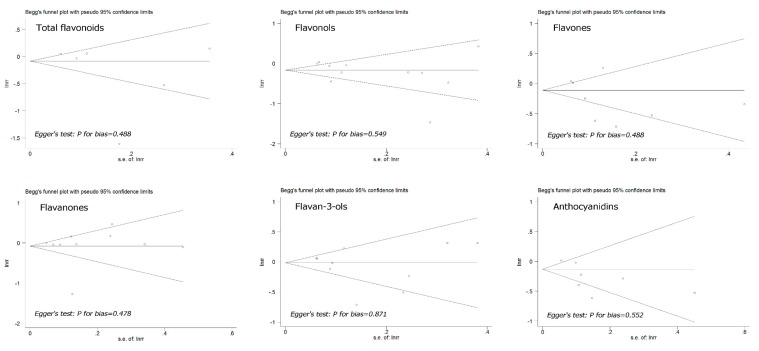
Begg’s funnel plot with pseudo-95% confidence limits for the RR of colorectal cancer and flavonoids intake (highest compared with lowest category of intake). Publication bias was evaluated with the use of funnel plots and with Egger’s regression asymmetry test (*p* < 0.1 was considered representative of statistically significant publication bias).

We performed Begg’s funnel plots and Egger’s tests to assess the publication bias in the included studies. As shown in Figure 3, the shape of the funnel plot did not reveal any evidence of obvious asymmetry. Egger’s test, which provides statistical evidence of the funnel plot symmetry, indicated little evidence of publication bias. Therefore, no significant publication bias was observed in these studies. 

## 4. Discussion

Despite the success of colonoscopy screening and recent advances in cancer treatment, colorectal cancer is still one of the most commonly diagnosed and deadly cancers, with a significantly increased incidence in developing countries where people are adapting to Western food and lifestyle. Colorectal cancer incidence rates are much higher in developed than in developing countries, which may be mainly attributed to the differences in diet and lifestyle. Studies have revealed that total energy, protein, fat, processed meat, alcohol, as well as fibre, omega-3 fatty acids, iron, and dietary acrylamide may significantly affect colorectal carcinogenesis and development [26,27]. Similarly, there is enough evidence to support an inverse association between the intake of dietary fruit, vegetables and tea and several cancers, including colorectal cancer [1,2,3]. Although the anti-carcinogenetic mechanism of these foods is unclear, the presence of dietary polyphenols may be one of the reasons. In the present study, our data indicated that high intake of dietary flavonols, flavones and anthocyanidins is associated with a decreased risk of colorectal cancer. However, substantial heterogeneities existed across these studies.

A meta-analysis study in 2016 showed no association between the incidence of colorectal cancer and total flavonoids, flavanones and flavan-3-ols, as well as dietary flavonols, flavones and anthocyanidins [28]. The summary risk estimate was 0.94 (95% CI 0.81–1.09), 1.05 (95% CI, 0.92–1.19), 0.90 (95% CI, 0.78–1.04) and 0.86 (95% CI, 0.71–1.03), 0.91 (95% CI, 0.78–1.05), 0.78 (95% CI, 0.61–1.01), respectively. The difference in the results between this study and ours is that the present up-to-date meta-analysis included more studies, especially articles published in the last 2 years. The previous meta-analysis included 6 studies for flavonols, 5 studies for flavones and 4 studies for anthocyanidins, while the present meta-analysis included 12 studies for flavonols, 8 studies for flavones and 7 studies for anthocyanidins. Grosso et al. provided a comprehensive review of existing observational studies on flavonoid and lignan intake and cancer risk [9]. The systematic analysis included 143 studies and showed only slight evidence of the association between dietary polyphenol intake and cancer risk. In particular, significant findings on flavonoids were rarely retrieved from prospective studies. Our study also indicated that associations were found only among case-control studies. The prospective cohort studies revealed no significant association between colorectal cancer risk and the intake of flavonoids.

Retrospective studies are susceptible to recall bias, so prospective cohort studies are assumed to have higher methodological quality. Nevertheless, case-control studies are also important in revealing risk and preventive factors for diseases, especially when experimental data and mechanism investigation confirm and support the findings. In fact, numerous studies in animals and cells have shown that some flavonoids in the diet can inhibit colon cell proliferation, minimize the effect of mutation, alleviate DNA oxidation damage, regulate phase I and phase II enzymes activities, suppress oncogene expression, modulate cell growth signalling pathways and mediate inflammatory responses in vitro and in vivo [29,30,31,32]. Furthermore, in a small clinical trial, long-term daily interventions with a flavonoid mixture (20 mg apigenin plus 20 mg epigallocatechin gallate) were shown to reduce the risk of colon cancer recurrence in patients with resected colon cancer [33]. 

Currently, the associations among diet, gut microbiota and colorectal cancer has drawn much attention in the area of cancer research [34,35]. Different diets could reshape the community structure of gut microbiota and influence its function by modulating the production of metabolites and consequently promoting or suppressing the tumourigenesis of colonic epithelial cells through cell metabolism, microbiota homeostasis, and anti-proliferative, immunomodulatory and genetic/epigenetic methods of regulation [36,37,38,39]. Studies have shown that non-absorbed flavonoids reach the colon in relatively high concentrations, particularly for polymeric flavonoids, where they are extensively metabolized by the gut microbiota to become small phenolic acids [40,41,42]. Investigating the associations among flavonoids, gut microbiota and colorectal cancer is of interest; some flavonoids may affect colorectal carcinogenesis and development via reactions with gut microbiota. 

It is important to note the limitations of the present meta-analysis. The results were mainly driven by case-control studies, and substantial heterogeneities existed across the included studies. Therefore, the findings may be promising but are inconclusive. Further prospective cohort studies using other methods to evaluate exposure (i.e., markers of consumption, metabolism, excretion) are needed to test the associations. Additionally, bioactive compounds in foods are complex and highly correlated. It is hard to tease apart their interaction and rule out the possibility that some components in food may be associated with flavonoids. Furthermore, gut microbiota may play important roles in flavonoid-induced chemoprevention, and different people with different gut microbiota might have different responses to the intake of flavonoids. Further studies are required to investigate the potential protective effects of various dietary flavonoids. 

## 5. Conclusions

In conclusion, our analyses supported the contention that several subclasses of flavonoids, flavonols, flavones and anthocyanidins may potentially decrease the risk of colorectal cancer. High intake of flavonols (such as quercetin) may reduce the risk of colon cancer, and high intake of flavones (such as apigenin) may reduce the risk of rectal cancer. More well-designed prospective cohort studies are needed to further investigate these associations.

## Figures and Tables

**Table 1:** Characteristics of the included studies.

Author, Year Region, Design, Cases/Controls	Flavonoids Exposure (mg/day), RR or OR and 95% CI				Adjustments
Cho YA 2017 Korean Case-control923/1846 [14]	Total flavonoids, MI (86.3)		Flavanones, MI (3.7)		Age, sex, BMI, education, total caloric intake, FHCC, and regular exercise.
	Q1 (<67.7)	1.00	Q1 (<1.16)	1.00	
	Q2 (67.7–98.3)	0.91 (0.72–1.14)	Q2 (1.16–3.54)	1.16 (0.90–1.50)	
	Q3 (98.3–141.7)	0.66 (0.52–0.85)	Q3 (3.54–8.99)	1.37 (1.07–1.76)	
	Q4 (≥141.7)	0.20 (0.14–0.28)	Q4 (≥8.99)	0.97 (0.74–1.27)	
	Flavonols, MI (16.4)		Flavan-3-ols, MI (10.2)		
	Q1 (<13.0)	1.00	Q1 (<5.62)	1.00	
	Q2 (13.0–19.2)	1.05 (0.84–1.32)	Q2 (5.62–13.19)	0.90 (0.71–1.14)	
	Q3 (19.2–27.4)	0.50 (0.39–0.65)	Q3 (13.19–30.38)	0.67 (0.52–0.87)	
	Q4 (≥27.4)	0.18 (0.13–0.25)	Q4 (≥30.38)	0.49 (0.38–0.66)	
	Flavones, MI (1.0)		Anthocyanidins, MI (17.0)		
	Q1 (<0.75)	1.00	Q1 (<11.4)	1.00	
	Q2 (0.75–1.12)	1.50 (1.17–1.92)	Q2 (11.4–18.9)	1.22 (0.96–1.56)	
	Q3 (1.12–1.70)	1.34 (1.04–1.73)	Q3 (18.9–29.7)	0.99 (0.77–1.26)	
	Q4 (≥1.70)	0.49 (0.36–0.67)	Q4 (≥29.7)	0.54 (0.40–0.71)	
Xu M 2016 China Case-control 1632/1632 [15]	Total flavonoids, MI (248.5)		Flavanones, MI (3.5)		Age, sex, marital status, education, income, occupation, family history of cancer, smoking status, passive smoking, alcohol, activity, BMI, and intakes of red and processed meat, poultry and fish, total dairy products and eggs.
	Q1	1.00	Q1	1.00	
	Q2	1.11 (0.90–1.38)	Q2	0.76 (0.62–0.92)	
	Q3	1.04 (0.84–1.31)	Q3	0.61 (0.50–0.76)	
	Q4	1.06 (0.85–1.32)	Q4	0.28 (0.22–0.36)	
	Flavonols, MI (34.5)		Flavan-3-ols, MI (26.6)		
	Q1	1.00	Q1	1.00	
	Q2	0.83 (0.66–1.03)	Q2	1.18 (0.94–1.47)	
	Q3	0.81 (0.65–1.00)	Q3	1.18 (0.95–1.48)	
	Q4	0.80 (0.64–0.99)	Q4	1.25 (0.99–1.56)	
	Flavones, MI (2.6)		Anthocyanidins, MI (18.9)		
	Q1	1.00	Q1	1.00	
	Q2	0.55 (0.44–0.68)	Q2	0.91 (0.73–1.12)	
	Q3	0.54 (0.44–0.67)	Q3	0.93 (0.75–1.15)	
	Q4	0.54 (0.43–0.67)	Q4	0.80 (0.64–1.00)	
Zamora-Ros R Spain 2013 Case-control 424/401 [16]	Total flavonoids, MI (198.0)		Flavanones, MI (17.1)		Sex, age, BMI, FHCC, energy intake, alcohol and fiber intake, red and processed meat intake, tobacco, PA, aspirin, NSAID.
	Q1 (<68.9)	1.00	Q1 (<3.7)	1.00	
	Q2 (68.9–108.9)	0.99 (0.66–1.49)	Q2 (3.7–9.1)	1.46 (0.97–2.19)	
	Q3 (108.9–167.9)	0.88 (0.57–1.37)	Q3 (9.1–17.7)	1.09 (0.71–1.66)	
	Q5 (>167.9)	0.59 (0.35–0.99)	Q5 (>17.7)	1.19 (0.75–1.91)	
	Flavonols, MI (14.5)		Flavan-3-ols, MI (15.2)		
	Q1 (<5.1)	1.00	Q1 (<4.9)	1.00	
	Q2 (5.1–8.3)	0.98 (0.65–1.47)	Q2 (4.9–8.1)	0.93 (0.61–1.40)	
	Q3 (8.4–11.5)	0.78 (0.50–1.23)	Q3 (8.2–12.9)	1.05 (0.69–1.61)	
	Q5 (>11.5)	0.79 (0.46–1.33)	Q5 (>12.9)	0.79 (0.49–1.28)	
	Flavones, MI (2.2)		Anthocyanidins, MI (11.4)		
	Q1 (<0.7)	1.00	Q1 (<3.3)	1.00	
	Q2 (0.7–1.2)	0.76 (0.51–1.15)	Q2 (3.3–6.5)	0.74 (0.49–1.12)	
	Q3 (1.3–2.1)	0.79 (0.52–1.21)	Q3 (6.6–10.6)	0.75 (0.50–1.14)	
	Q5 (>2.1)	0.59 (0.37–0.93)	Q5 (>10.6)	0.75 (0.47–1.20)	
Djuric Z USA 2012 Case-control 1163/1501 [17]	Flavonol (quercetin), MI (8.35)				Age, gender, PA, BMI, FHCC, education, NSAID, total fat, fiber, carotenoids, folate.
	Q1 (<4.3)	1.00			
	Q2 (4.3–6.3)	0.80 (0.64–1.01)			
	Q3 (6.3–10.3)	1.06 (0.84–1.33)			
	Q5 (>10.3)	0.96 (0.76–1.22)			
Kyle JA UK 2010 Case-control 264/408 [18]	Flavonols, MI (30.1)		Flavanones, MI (15.6)		Energy, age at diagnosis, family history, NSAID, aspirin, Mn, riboflavin, vitamin C, folate.
	Q1 (<19.3)	1.00	Q1 (<2.7)	1.00	
	Q2 (19.3–30.4)	1.0 (0.6–1.7)	Q2 (2.7–13.4)	1.5 (0.9–2.5)	
	Q3 (30.4–40.4)	1.3 (0.8–2.1)	Q3 (13.4–32.2)	1.4 (0.9–2.4)	
	Q5 (>40.4)	0.8 (0.5–1.3)	Q5 (>32.2)	1.6 (1.0–2.6)	
	Flavan-3-ols, MI (127.8)				
	Q1 (<67.1)	1.00			
	Q2 (67.1–119.2)	0.7 (0.4–1.1)			
	Q3 (119.2–188.8)	1.3 (0.8–2.2)			
	Q5 (>188.8)	0.6 (0.4–1.0)			
Theodoratou E Scotland 2007 Case-control 1456/1456 [19]	Flavonols, MI (27.4)		Flavanones, MI (20.4)		Total energy, FHCC, fiber intake, alcohol, NSAID intake, smoking, BMI, and PA, fruit, vegetable intake.
	Q1 (<16.0)	1.00	Q1 (<16.7)	1.00	
	Q2 (16.1–27.4)	0.57 (0.43–0.76)	Q2 (16.7–32.7)	1.43 (1.15–1.80)	
	Q3 (27.5–36.8)	0.41 (0.27–0.63)	Q3 (32.7–45.2)	1.35 (1.08–1.70)	
	Q4 (>36.8)	0.23 (0.13–0.40)	Q5 (>45.2)	1.18 (0.93–1.50)	
	Flavones, MI (1.0)		Flavan-3-ols, MI (115.4)		
	Q1 (<0.5)	1.00	Q1 (<42.6)	1.00	
	Q2 (0.5–1.1)	1.05 (0.85–1.31)	Q2 (42.6–115.3)	1.10 (0.81–1.49)	
	Q3 (1.1–1.9)	1.01 (0.81–1.26)	Q3 (115.3–162.1)	1.56 (0.98–2.50)	
	Q4 (>1.9)	1.30 (1.01–1.68)	Q5 (>162.1)	1.37 (0.73–2.57)	
Rossi M Italy 2006 Case-control 1953/4154 [20]	Total flavonoids, MI (137.8)		Flavanones, MI (38.3)		Age, sex, study center, FHCC, education, alcohol consumption, BMI, occupational PA, and energy intake.
	Q1 (<75.3)	1.00	Q1 (<12.5)	1.00	
	Q2 (75.4–108.5)	0.90 (0.75–1.08)	Q2 (12.6–28.7)	0.88 (0.74–1.05)	
	Q3 (108.6–141.6)	0.79 (0.66–0.94)	Q3 (28.8–35.5)	0.89 (0.75–1.07)	
	Q4 (141.7–191.1)	0.81 (0.67–0.97)	Q4 (35.6–67.0)	0.80 (0.67–0.96)	
	Q5 (>191.1)	0.97 (0.81–1.16)	Q5 (>67.0)	0.96 (0.81–1.15)	
	Flavonols, MI (21.6)		Flavan-3-ols, MI (54.0)		
	Q1 (<13.2)	1.00	Q1 (<20.8)	1.00	
	Q2 (13.3–17.3)	0.80 (0.67–0.95)	Q2 (20.9–34.4)	0.75 (0.63–0.91)	
	Q3 (17.4–22.0)	0.77 (0.64–0.91)	Q3 (34.5–51.7)	0.75 (0.62–0.90)	
	Q4 (22.1–28.5)	0.74 (0.62–0.88)	Q4 (51.8–88.5)	0.79 (0.65–0.95)	
	Q5 (>28.6)	0.64 (0.54–0.77)	Q5 (>88.5)	0.98 (0.82–1.18)	
	Flavones, MI (0.5)		Anthocyanidins, MI (20.0)		
	Q1 (<0.3)	1.00	Q1 (<5.3)	1.00	
	Q2 (0.3–0.4)	0.82 (0.69–0.98)	Q2 (5.4–11.5)	0.81 (0.68–0.96)	
	Q3 (0.4–0.5)	0.72 (0.61–0.86)	Q3 (11.6–19.4)	0.78 (0.65–0.93)	
	Q4 (0.5–0.7)	0.76 (0.64–0.91)	Q4 (19.5–31.7)	0.64 (0.53–0.77)	
	Q5 (>0.7)	0.78 (0.65–0.93)	Q5 (>31.7)	0.67 (0.54–0.82)	
Zamora-Ros R Europe 2017 Prospective cohort 4517(477,312) [21]	Total flavonoids, MI (418)		Flavanones, MI (40)		Sex, age, center, smoking, PA, education, BMI, total energy, alcohol, red and processed meat, fibre and calcium intakes, menopausal status, hormone replacement therapy use, contraceptive use.
	Q1(<223)	1.00	Q1 (<8.2)	1.00	
	Q2 (223–346)	1.09 (0.99–1.20)	Q2 (8.2–18.1)	0.96 (0.88–1.05)	
	Q3 (347–507)	1.10 (0.99–1.22)	Q3 (18.2–33.3)	0.99 (0.91–1.09)	
	Q4 (508–771)	1.07 (0.96–1.20)	Q4 (33.4–65.9)	0.95 (0.86–1.05)	
	Q5 (>771)	1.05 (0.93–1.18)	Q5 (>65.9)	1.00 (0.91–1.10)	
	Flavonols, MI (28)		Flavan-3-ols, MI (325)		
	Q1 (<13.9)	1.00	Q1 (<135)	1.00	
	Q2 (13.9–23.0)	1.02 (0.92–1.13)	Q2 (135–228)	1.08 (0.98–1.19)	
	Q3 (23.1–34.8)	1.03 (0.93–1.15)	Q3 (229–356)	1.15 (1.04–1.28)	
	Q4 (34.9–61.7)	0.99 (0.88–1.11)	Q4 (357–584)	1.10 (0.99–1.23)	
	Q5 (>61.7)	1.00 (0.89–1.14)	Q5 (>584)	1.05 (0.93–1.19)	
	Flavones, MI (9.3)		Anthocyanidins, MI (25)		
	Q1 (<5.1)	1.00	Q1 (<10.3)	1.00	
	Q2 (5.1–7.8)	1.01 (0.93–1.11)	Q2 (10.3–19.1)	0.95 (0.87–1.04)	
	Q3 (7.9–11.0)	0.94 (0.85–1.03)	Q3 (19.2–32.5)	0.96 (0.87–1.06)	
	Q4 (11.1–16.5)	1.04 (0.94–1.15)	Q4 (32.6–58.9)	1.00 (0.91–1.10)	
	Q5 (>16.5)	1.04 (0.92–1.17)	Q5 (>58.9)	1.01 (0.91–1.13)	
Nimptsch K 2016 USA Prospective cohort 2519(118,842) [22]	Flavonols, MI (-)		Flavanones, MI (-)		Age, smoking, history of colorectal cancer, history of endoscopy, regular aspirin use, BMI, PA, alcohol, total calories, vitamin D, total calcium, red meat, and processed meat intake.
	Q1 (9.6)	1.00	Q1 (23.0)	1.00	
	Q2	0.92 (0.78–1.09)	Q2	0.99 (0.82–1.19)	
	Q3 (15.2)	0.92 (0.81–1.05)	Q3 (52.0)	1.05 (0.86–1.28)	
	Q4	1.10 (0.97–1.24)	Q4	0.99 (0.79–1.23)	
	Q5 (31.9)	1.04 (0.91–1.18)	Q5 (56.6)	0.96 (0.84–1.10)	
	Flavones, MI (-)		Flavan-3-ols, MI (-)		
	Q1 (1.4)	1.00	Q1 (10.2)	1.00	
	Q2	0.99 (0.87–1.13)	Q2	0.96 (0.84–1.09)	
	Q3 (2.6)	1.02 (0.89–1.16)	Q3 (25.0)	0.98 (0.86–1.11)	
	Q4	1.00 (0.88–1.14)	Q4	1.00 (0.87–1.15)	
	Q5 (2.8)	1.01 (0.89–1.15)	Q5 (141.8)	1.07 (0.95–1.21)	
	Anthocyanidins, MI (-)				
	Q1 (5.5)	1.00			
	Q2	0.93 (0.82–1.05)			
	Q3 (14.6)	1.04 (0.92–1.18)			
	Q4	0.97 (0.85–1.10)			
	Q5 (23.6)	0.98 (0.81–1.19)			
Simons CC Netherland 2009 Prospective cohort 2485(120,852) [23]	Flavonols, MI (26.8) men		Flavonols, MI (28.9) women		Age, FHCC, smoking, alcohol, PA, BMI and processed meat intake.
	Q1 (<16.0)	1.00	Q1 (<18.4)	1.00	
	Q2 (16.0–22.5)	0.95 (0.75–1.21)	Q2 (18.4–25.0)	0.85 (0.66–1.10)	
	Q3 (22.5–28.3)	0.81 (0.63–1.04)	Q3 (25.0–31.1)	0.98 (0.76–1.25)	
	Q3 (28.3–36.1)	0.89 (0.70–1.14)	Q3 (31.1–38.4)	0.80 (0.62–1.03)	
	Q5 (>36.1)	0.97 (0.76–1.23)	Q5 (>38.4)	0.90 (0.70–1.16)	
	Flavan-3-ols, MI (58.6) men		Flavan-3-ols, MI (66.2) women		
	Q1 (<24.2)	1.00	Q1 (<36.2)	1.00	
	Q2 (24.2–44.4)	1.01 (0.79–1.28)	Q2 (36.2–51.6)	0.90 (0.70–1.16)	
	Q3 (44.4–62.8)	0.85 (0.67–1.09)	Q3 (51.6–75.4)	0.79 (0.61–1.02)	
	Q3 (62.8–84.4)	0.85 (0.67–1.08)	Q3 (75.4–95.9)	1.02 (0.79–1.30)	
	Q5 (>84.4)	0.99 (0.77–1.25)	Q5 (>95.9)	0.79 (0.61–1.02)	
Mursu J Finland 2008 Prospective cohort 55(2590) [24]	Total flavonoids, MI (131.0)		Flavanones, MI (2.9)		Age, examination years, BMI, smoking, PA, intakes of alcohol, total fat, saturated fat, fiber, vitamin C and E.
	Q1 (9.1)	1.00	Q1	1.00	
	Q2 (16.3)	0.74 (0.34–1.60)	Q2	0.84 (0.36–1.98)	
	Q3 (82.7)	0.52 (0.22–1.23)	Q3	1.80 (0.85–3.85)	
	Q5 (416.3)	1.16 (0.58–2.34)	Q4	0.90 (0.37–2.20)	
	Flavonols, MI (9.5)		Flavan-3-ols, MI (112.3)		
	Q1	1.00	Q1	1.00	
	Q2	0.68 (0.30–1.58)	Q2	1.04 (0.48–2.28)	
	Q3	0.86 (0.38–1.97)	Q3	0.80 (0.34–1.86)	
	Q4	1.53 (0.72–3.23)	Q4	1.37 (0.65–2.89)	
	Flavones, MI (0.3)		Anthocyanidins, MI (5.9)		
	Q1	1.00	Q1	1.00	
	Q2	1.26 (0.59–2.68)	Q2	0.69 (0.30–1.60)	
	Q3	1.16 (0.54–2.50)	Q3	1.62 (0.80–3.31)	
	Q4	0.71 (0.30–1.65)	Q4	0.59 (0.24–1.41)	
Knekt P Finland 2002 Prospective cohort 90(9865) [25]	Flavonol (quercetin), MI (3.3)		Flavanone (hesperetin), MI (15.1)		Sex, age, geographic area, occupation, smoking, and BMI.
	Q1 (<1.7)	1.00	Q1 (<1.6)	1.00	
	Q2 (1.7–2.7)	0.84 (0.48–1.49)	Q2 (1.6–10.2)	1.49 (0.87–2.58)	
	Q3 (2.7–3.4)	0.97 (0.56–1.70)	Q3 (10.2–20.9)	1.56 (0.86–2.84)	
	Q5 (>3.4)	0.62 (0.33–1.17)	Q5 (>20.9)	0.97 (0.50–1.90)	

CI: confidence intervals; MI: median intake; PA: physical activity; FHCC: family history of colorectal cancer; BMI: body mass index; NSAID: non-steroidal anti-inflammatory drug.

**Table 2:** Summary risk estimates for dietary flavonoids intake (highest vs. lowest) and colorectal cancer risk.

Study		No. of Studies	RR(95% CI)	Heterogeneity Test	
				*p*	*I*^2^ (%)
**Flavonols**					
**Design**	Case-control	7	0.54 (0.35–0.84)	0.00	93.1
	Prospective	5	1.00 (0.92–1.08)	0.369	6.6
**Cancer type**	Colon	7	0.80 (0.68–0.94)	0.025	58.4
	Rectum	7	0.93 (0.74–1.18)	0.009	64.8
**Population**	European	8	0.75 (0.58–0.96)	0.00	83.3
	Asian	2	0.51 (0.42–0.61)	0.00	98.2
	USA	2	1.02 (0.91–1.14)	0.561	0.0
**Flavones**					
**Design**	Case-control	5	0.73 (0.65–0.81)	0.00	88.4
	Prospective	3	1.02 (0.94–1.11)	0.665	0.0
**Cancer type**	Colon	4	0.88 (0.69–1.13)	0.011	73.1
	Rectum	4	0.82 (0.70–0.97)	0.608	0.0
**Population**	European	5	0.91 (0.72–1.16)	0.002	76.5
	Asian	2	0.52 (0.44–0.63)	0.618	0
	USA	1	1.01 (0.89–1.15)	-	-
**Anthocyanidins**					
**Design**	Case-control	4	0.69 (0.60–0.78)	0.196	36
	Prospective	3	1.00 (0.91–1.10)	0.488	0.0
**Cancer type**	Colon	3	0.81 (0.58–1.12)	0.022	73.7
	Rectum	3	0.84 (0.59–1.21)	0.099	56.7
**Population**	European	4	0.91 (0.83–1.00)	0.004	77.6
	Asian	2	0.66 (0.45–0.98)	0.034	77.7
	USA	1	0.98 (0.81–1.19)	-	-
